# Hidden Appendicoliths and Their Impact on the Severity and Treatment of Acute Appendicitis

**DOI:** 10.3390/jcm13144166

**Published:** 2024-07-16

**Authors:** Maximilian Dölling, Mirhasan Rahimli, Jonas Pachmann, Malik Szep, Sara Al-Madhi, Mihailo Andric, Ulf D. Kahlert, Tobias Hofmann, Michael Boettcher, Luis E. Muñoz, Martin Herrmann, Aristotelis Perrakis, Roland S. Croner

**Affiliations:** 1 Department of General, Visceral, Vascular and Transplant Surgery, University Hospital Magdeburg, 39120 Magdeburg, Germany; 2 Molecular and Experimental Surgery, Department of General, Visceral, Vascular and Transplant Surgery, Faculty of Medicine, University Hospital Magdeburg, Otto-von-Guericke University, 39120 Magdeburg, Germany; 3 Central Emergency Department, University Hospital Magdeburg, Otto-von-Guericke-University, 39120 Magdeburg, Germany; 4 Department of Pediatric Surgery, University Medical Center Mannheim, University of Heidelberg, 68167 Mannheim, Germany; 5 Department of Internal Medicine 3—Rheumatology and Immunology, Universitätsklinikum Erlangen, Friedrich-Alexander Universität Erlangen-Nürnberg (FAU), 91054 Erlangen, Germany; 6 Deutsches Zentrum für Immuntherapie (DZI), Universitätsklinikum Erlangen, Friedrich-Alexander Universität Erlangen-Nürnberg (FAU), 91054 Erlangen, Germany; 7 Iatriko Medical Center, Department of General, Minimally Invasive Surgery and Surgical Oncology, 15125 Athens, Greece

**Keywords:** acute appendicitis, appendicoliths, faecoliths, perforated appendicitis

## Abstract

**Background/Objectives**: In patients diagnosed with uncomplicated acute appendicitis (UAA), the absence of calcified deposits or stones, called appendicoliths, often leads to consideration of non-operative treatment (NOT), despite the notable treatment failure rate associated with this approach. Previous research has indirectly estimated the prevalence of appendicoliths to range between 15% and 38% retrospectively by CT scan, intraoperative palpation, and pathology report, thereby potentially missing certain concrements. Our hypothesis proposes that this reported prevalence significantly underestimates the occurrence of appendicoliths, which could explain the high failure rate of 29% of patients with appendicitis observed with NOT. **Methods**: In our prospective study, conducted with a cohort of 56 adult patients diagnosed with acute appendicitis (AA), we employed intraoperative extracorporeal incisions of the vermiform appendix, in addition to standard diagnostic methods. **Results**: Our findings revealed 50% more appendicoliths by intraoperative incision (*n* = 36, *p* < 0.001) compared to preoperative imaging (*n* = 24). Appendicoliths were present in 71.4% (*n* = 40, *p* < 0.001) of AA patients. **Conclusions**: These results suggest that conventional diagnostic procedures plausibly underestimate the actual prevalence of appendicoliths, potentially elucidating the frequent treatment failures observed in NOT approaches applied to patients with UAA.

## 1. Introduction

Acute appendicitis (AA) is a common emergency in general surgery with an annual worldwide incidence of 228/100,000 [1]. The lifetime risk of AA is 8.6% in men and 6.7% in women with the peak prevalence in adolescents between 10 and 14 years in men and 15 and 19 years in women, respectively [2,3]. The gold standard of treatment of AA is surgical appendectomy, even though non-operative treatment (NOT) is a controversial alternative for mild clinical courses [4,5,6]. Traditionally, AA has been classified by pathologic findings as catarrhalic, ulcerophlegmonous, gangrenous, and perforated. Because of heterogenous concepts of classification and treatment of AA worldwide, the first Consensus Conferences on AA were held 2015 in Jerusalem (WSES, updated 2020) and Bucharest (EAES) in order to develop international guidelines on this topic [5]. According to the EAES guidelines, patients with AA are classified into uncomplicated (UAA) and complicated (CAA) acute appendicitis [5,7]. Depending on the diagnosis of UAA or CAA, patients receive a (laparoscopic) appendectomy or are considered for conservative anti-infective therapy regimes [4,5,8]. However, factors that determine the therapy for patients with AA are still not consistent, leading to a failure rate of NOT of about 29% within 90 days [5,6,9].

Even though AA is a very common disease with the necessity of surgical intervention, the pathophysiology of the disease is still elusive [10,11,12]. Moreover, it has also been proposed that there are two entities of appendicitis: one that leads to perforation and one that resolves by itself [11,13]. It has been speculated that the two entities of disease may depend on different pathophysiology. In most cases, the obstruction of the appendicular lumen is caused by appendicoliths, especially those located at the base of the appendix, with a higher risk of perforation [14,15], but in rare cases, luminal obstruction may be caused by neuroendocrine tumors [16], intestinal parasitic infestations such as Enterobius vermicularis [17], or foreign bodies [18]. Furthermore, viral infection can cause mucosal ulcerations and secondary bacterial invasion [10,19]. The theory of luminal obstruction is supported by clinical data that identify appendicoliths as independent prognostic factors for the failure of NOT in UAA and a risk factor for perforation [11]. Also, appendicoliths were identified being a risk factor for prolonged hospital stay [20]. For this reason, guidelines for the treatment of AA included the presence of appendicoliths as a reason for performing an appendectomy [21,22,23].

Up to now, several studies have estimated the prevalence of appendicoliths in AA by using CT scan combined with ultrasound [24,25]. They estimated the prevalence of appendicoliths in adults with AA to be 27.0–38.7% [4,25]. The CODA study in 2020, an RCT with 1552 adults with AA, compared antibiotic therapy with appendectomy and revealed a therapy failure of antibiotic treatment within a 90-day interval in 41% of the patients that showed appendicoliths in imaging [4,26,27]. Patients receiving antibiotic therapy without appendicoliths in imaging had a therapy failure rate of only 25% (overall 29%) [4]. It has been shown that the presence of appendicoliths is a strong predictor for the failure of NOT; however, the reason for therapy failure of patients without appendicoliths is still unclear [28,29]. A possible explanation would be that non-calcified appendicoliths escape detection because they do not show sufficient contrast on imaging. Studies that combined different diagnostic procedures including imaging, intraoperative palpation of removed vermiform appendix, and histopathological findings estimated the prevalence of appendicoliths in patients with AA with 18–52% [9,30,31].

However, these studies did not directly investigate the appendicular content. This is important, as after fixation in formaldehyde, non-calcified appendicoliths might dissolve before histopathological examination. Currently there are no studies which directly measured the prevalence of appendicoliths before fixation. We therefore hypothesize that the prevalence of appendicoliths in patients with AA was underestimated by current diagnostic methods [32]. Furthermore, there is only limited information about the influence of the size of appendicoliths as a potential cause for luminal obstruction on the outcome of AA. Thus, we hypothesize that the diameters of the appendicoliths correlate with the outcome. Therefore, we use direct intraoperative extracorporeal examination of appendicular lumina to determine the prevalence of appendicoliths in patients with AA. In addition, we measured the sizes of appendicoliths and analyzed their influence on the severity of AA.

## 2. Materials and Methods

### 2.1. Patients

All patients over the age of 18 years who were admitted to the University Hospital Magdeburg, Saxony-Anhalt, Germany, intraoperatively diagnosed with “acute appendicitis”, gave informed consent, and given surgical removal and intraoperative examination by extracorporeal incision of vermiform appendix from July 2022 until October 2023 were included in the study (*n* = 56) (Supplementary Figure S1). Ten patients were excluded because they received conservative antibiotic treatment. Two patients were excluded due to false positive intraoperative diagnosis of acute appendicitis as confirmed by histopathology. Simultaneous appendectomies as a part of extended surgery such as gynecological operations, hemicolectomy, ileocecal resection, and extended bowel resection were not considered. The sample size calculation was performed using preliminary data due to the lack of existing data for the novel technique to estimate the prevalence of appendicoliths in patients with acute appendicitis (AA) undergoing surgery. Given an effect size of 19.2%, a statistical power of 80%, an alpha level of 0.05, and two-sided testing, we estimated the necessary sample size for achieving significance to be 50 patients (Supplementary Figure S2).

### 2.2. Outcomes

Primary outcome was the prevalence of appendicoliths. Secondary outcomes were appendicolith measurements (number of appendicoliths within a specimen, cumulative volume of all appendicoliths within a specimen, diameter of the largest appendicolith within a specimen, volume of the largest appendicolith within a specimen), disease severity defined by uncomplicated acute appendicitis (UAA) and complicated acute appendicitis (CAA), simple appendicitis (catarrhalic and ulcerophlegmonous appendicitis) and advanced appendicitis (gangrenous and perforated appendicitis with or without perityphlitic abscess), and hospitalization time.

### 2.3. Definitions, Multilevel Assessment of the Presence of Appendicoliths, and Data Collection

Sticking to Sex and Gender Equity in Research (SAGER) Guidelines, we reported differences in gender by self-reporting of sex assigned at birth.

Appendicoliths were defined as fecal concretions or pellets with or without calcification found within the vermiform appendix. The presence of appendicoliths is defined as identification of appendicoliths by at least one out of three different methods: pre-operative imaging, intraoperative visualization of concretions after incision of vermiform appendix, and histopathological report. According to EAES guidelines, we defined UAA as catarrhalic AA and CAA as ulcerophlegmonous, gangrenous, or perforated AA including perityphilitic abscess based on intraoperative diagnosis and confirmation by pathologists. Preoperative imaging included computer tomography (layers: 1 mm, intravenous application of contrast media) and standardized ultrasound. Image analysis and judgment about the presence of appendicoliths were performed by at least one qualified radiologist and reviewed by one attendant of radiology. In case of suspected malignancy, no intraoperative incision was performed, and the case was excluded from the study.

**Figure 1:**
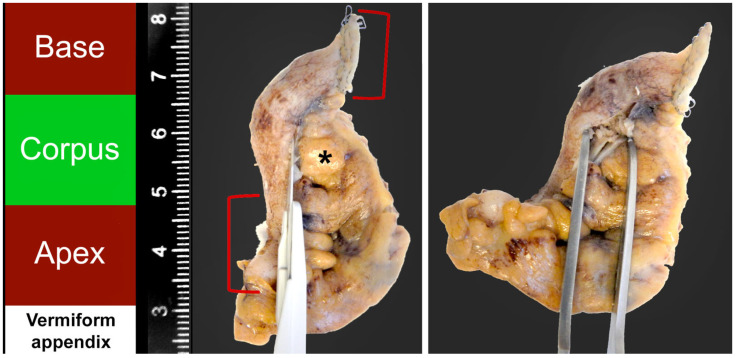
Manual of intraoperative examination of vermiform appendix shown on a formalin-fixed specimen. (1) Identification of basis (red) with stapler line, corpus (green) and apex (red) of vermiform appendix. (2) Incision along mesenteriolum (*) in the corpus. (3) Inspection and (4) rinsing with 0.9% sodium chloride of luminal side of vermiform appendix. Intraoperative incision on specimen before fixation with formalin according to this manual.

Intraoperatively, after removal from abdominal cavity, a longitudinal incision (length 4–6 cm) of the appendix parallel to the mesenteriolum was conducted and appendicular lumen was rinsed with 0.9% sodium chloride solution (Figure 1). Appendicoliths were collected, cleaned, and stored in 70% ethanol solution. Histopathological analysis on the presence of appendicoliths in the specimen was performed by an attendant pathologist and documented in the report. Collected appendicoliths were photographed, measured in three dimensions, and the volumina were calculated. Also, CT images were analyzed and appendicular volumina were calculated. Clinical data and patient information were collected from an electronic clinical record system including age, sex, admission date, laboratory values (C-reactive protein, leucocyte count), severity of disease, complications, date of discharge, and readmissions.

### 2.4. Group Allocation and Group Characteristics

According to the presence of appendicoliths patients were allocated to the groups “no appendicolith” and “appendicolith”. The presence of appendicoliths was assumed if one of the three modalities could identify an appendicolith. Absence of appendicoliths was assumed if none of the three modalities could identify an appendicolith. We analyzed patient characteristics, perioperative parameters, measures of appendicoliths, and follow-up data of entire cohort and between the two groups (patients with or without appendicoliths).

### 2.5. Statistical Analysis

Patient data were collected using Microsoft Excel (v16.79.1, Microsoft, Redmond, WA, USA) and statistically analyzed by using the statistical package STATA (v17.0, StataCorp, College Station, TX, USA). Graphs were created with GraphPad PRISM (v9, GraphPad Software, Inc., Boston, MA, USA). For reproducibility, the complete statistical analysis was logged. Non-parametric variables were analyzed with contingency tables and tested by chi-squared test statistics. We applied Shapiro–Wilk tests and evaluation of histograms for evaluation of normal distribution prior to testing of continuous variables. Additionally, variance homogeneity was analyzed by F-tests or Levene’s robust test in case of non-normality. When test assumptions were met, unpaired t-tests on continuous outcomes of dichotomous predictor variables were performed. For skewed distributions Wilcoxon–Mann–Whitney tests were applied. We used the number of cases and percentages (%) or means and standard deviations (SD) for presenting the qualitative and quantitative data, respectively. In case of non-normal distribution, we used median and IQR. To all statistical tests a significance level with a critical *p*-value of 0.05 with two-sided testing was applied. Logistic regression was used to detect predicting factors for complicated and advanced appendicitis. In a bidirectional stepwise strategy, parameters without a significant effect on the overall models were excluded. ROC regression analyses were applied for presentation of the models and the area under the curves (AUCs) were calculated. By calculating the Youden index, the cutoff value for the diameter of largest appendicoliths with maximum of sensitivity and specificity was identified.

## 3. Results

### 3.1. Population and Primary Outcome

From July 2022 to November 2023, a total of 56 patients with acute appendicitis (AA) were assessed for the presence of appendicoliths by a multistep procedure comprising imaging diagnostics (standardized ultrasound, computer tomography), intraoperative extracorporeal incision (Figure 1) with immediate examination of vermiform appendix, and histopathological examination. Table 1 displays the basic patient characteristics of the study population. Of 56 patients, 49 patients (87.5%) suffered from complicated acute appendicitis (CAA) compared to 7 patients (12.5%) of uncomplicated acute appendicitis (UAA). Patients were divided into an appendicolith positive (*n* = 40) and negative (*n* = 16) arm based on our combined multistep procedure. Both groups were similar in sex, age, BMI, ASA score, and leucocyte count (*p* > 0.05) (Table 1). There was a tendency of higher CRP in patients with appendicoliths compared to those without (70.9 *±* 78.8 versus 42.1 *±* 43.2 mg/l; *p* = 0.32).

### 3.2. Multilevel Assessment of the Presence of Appendicoliths in Patients with AA

Appendicoliths were detected by ultrasound, CT scan, intraoperative extracorporeal incision and examination, and histopathological examination. The detection rates differed depending on diagnostic modality (Table 2). After admission to hospital because of typical symptoms of AA, ultrasound and/or CT scans were performed. The intraoperative extracorporeal incision and examination of appendicular content revealed 36 appendicoliths (64.3%; *p* = 0.001). In a CT scan and histopathological examination, two appendicoliths (3.6%; *p* = 0.36) were found, which were missed by the intraoperative examination, respectively. Combined procedures yielded a detection rate of 40 out of 56 (71.4%). Intraoperative incision increased the sensitivity for the detection of appendicoliths compared to preoperative imaging from 60% (24 of 40 cases) to 90% (36 of 40 cases, *p* = 0.008).

### 3.3. Characteristics of Appendicoliths Differ between UAA and CAA

**Figure 2:**
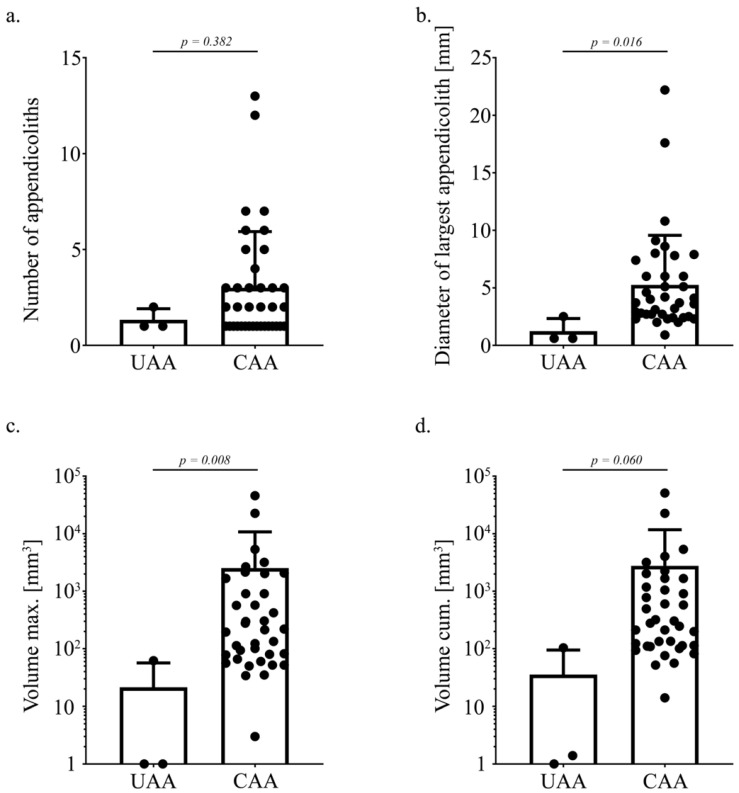
Comparison of appendicoliths between uncomplicated and complicated acute appendicitis (UAA and CAA). (**a**) The number of appendicoliths within a specimen was similar in UAA and CAA (*p* = 0.382). (**b**,**c**) The diameter of the largest appendicolith in mm and the volume of the largest appendicolith in mm^3^ were higher in CAA patients compared to UAA patients (*p* = 0.016 and *p* = 0.008, respectively). (**d**) The cumulative volume of appendicoliths calculated by the summation of the volume of each appendicolith within a specimen showed a tendency to be higher in patients with CAA compared to UAA (*p* = 0.060).

Imaging data and collected appendicoliths from surgery were analyzed for the number of appendicoliths, the diameter/volume of the largest appendicolith (Volume max.), and the cumulative volume (Volume cum.) of all appendicoliths found within a specimen (Figure 2a–d; Supplementary Table S1). In patients with CAA, the average number (median = 2; IQR: 1; 3; *p* = 0.382; Figure 2a) of appendicoliths within a specimen did not show a relevant difference in comparison to patients with UAA. However, the diameter of the largest appendicoliths was higher in patients with CAA (median = 3.7 mm^3^; IQR: 2.5 mm^3^, 6.0 mm^3^; *p* = 0.016; Figure 2b). Furthermore, the maximal volume of the largest appendicoliths was higher in CAA (median = 216.5 mm^3^; IQR: 72 mm^3^, 1288 mm^3^; *p* = 0.008; Figure 2c). The cumulative volume of all appendicoliths showed a tendency to be increased in patients with CAA (median = 304 mm^3^; IQR: 111 mm^3^, 1664 mm^3^; *p* = 0.060; Figure 2d).

### 3.4. The Diameter of Appendicoliths Predict the Severity of AA

**Figure 3:**
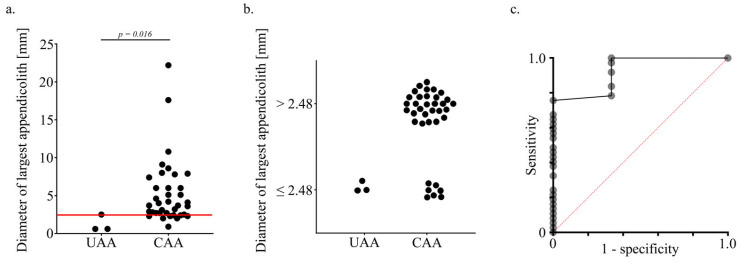
Diameter of appendicoliths in uncomplicated and complicated acute appendicitis (UAA and CAA). (**a**) The distribution plot depicts the difference between the diameters of the largest appendicoliths between UAA and CAA (*p* = 0.016). The logistic regression analysis revealed a cutoff for the predictive value of appendicoliths at maximum at 2.48 mm indicated by the red line. (**b**) Distribution of appendicoliths based on the cutoff value of 2.48 mm with either UAA or CAA. Some patients with CAA had appendicoliths < 2.48 mm reflecting mostly patients with ulcerophlegmonous appendicitis. (**c**) The Youden index of the ROC curve for the diameter of the largest appendicoliths and CAA revealed a high predictive value with an AUC = 0.93 at a cutoff of 2.48 mm.

Next, a logistic regression model was applied to investigate the predictive power of the characteristics of appendicoliths for the development of CAA (Supplementary Table S2). We adjusted the model in a bidirectional stepwise strategy leaving over the diameter of appendicoliths as a single predictor for CAA (Pseudo R^2^ = 0.499; p_model_ = 0.001; p_diameter_ = 0.025). Optimizing sensitivity and specificity in the same model, we found appendicoliths to be predictive at the cut point diameter of 2.48 mm for patients developing a CAA (AUC = 0.93; sensitivity 78.4%; specificity 100%; CI_95%_: 0.62; 0.90) (Figure 3a–c).

### 3.5. Appendicoliths Lead to Advanced Stages of CAA and Longer Hospitalization

**Figure 4:**
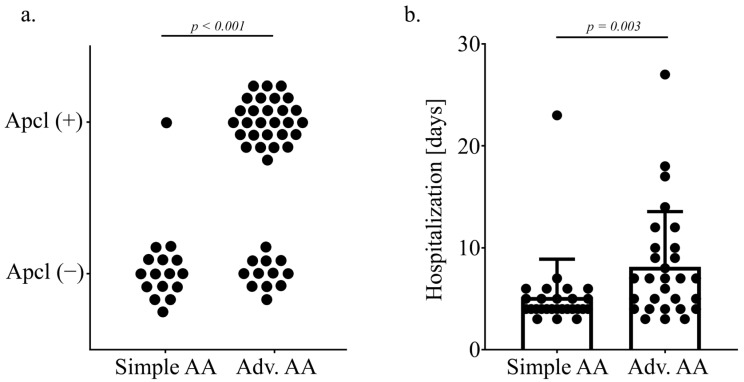
Comparison of the presence of appendicoliths and hospitalization time in simple and advanced AA. (**a**) In patients with advanced stages of AA (gangrenous and perforation with and without abscess), more appendicoliths were found (*p* < 0.001). (**b**) The hospitalization of patients with advanced AA (median = 7.0 days) was increased compared to patients with simple AA (median = 4.0 days; *p* = 0.003).

To examine the impact of appendicoliths on more advanced stages of AA the population was divided into patients with catarrhalic and ulcerophlegmonous appendicitis, called simple AA (*n* = 27), and patients with gangrenous and perforated appendicitis with or without abscess, called advanced AA (*n* = 29) (Table 3). The analysis showed a higher proportion of appendicoliths in patients with advanced AA compared to patients with simple AA (*p* < 0.001) (Figure 4a). In seven out of eight cases with gangrenous appendicitis, appendicoliths were found. Overall, 37.5% of patients with appendicoliths showed the intraoperative picture of perforated appendicitis with or without perityphilitic abscess. There was no perforated appendicitis without appendicoliths. Furthermore, the mean hospitalization of patients with appendicoliths is longer than in those without appendicoliths (8.14 days *±* 5.4 days vs. 5.19 days *±* 3.7 days; *p* = 0.003) (Figure 4b). There was no relevant difference in major surgical complications between both groups (*p* = 0.124).

**Figure 5:**
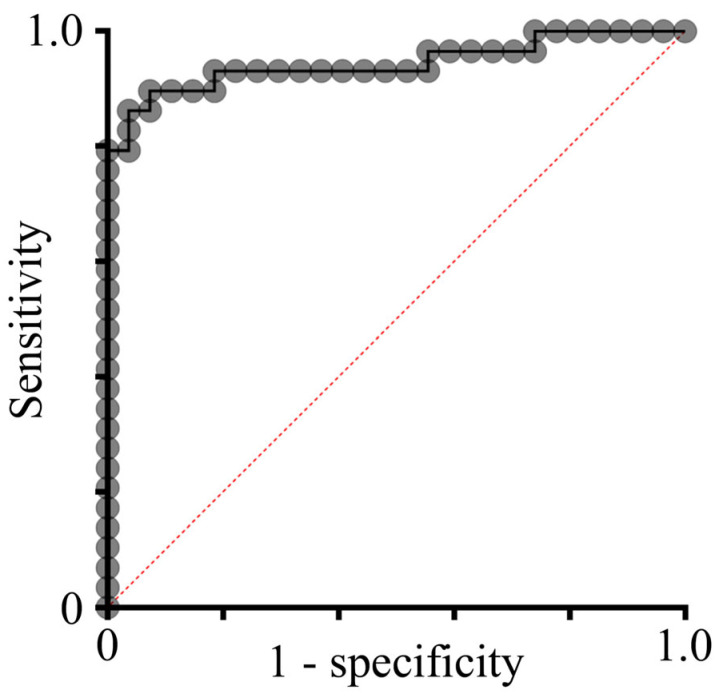
ROC curve of the prediction of advanced AA including appendicoliths, BMI, ASA score, and CRP. ROC analysis of multiple logistic regression model shows a high predictive power of appendicoliths, BMI, ASA score, and CRP for advanced stages of AA (AUC = 0.955).

To find an optimal model for the prediction of advanced stages of disease, we performed a multiple logistic regression analysis with the selection of optimal predictors (Table 4). Applying a bidirectional elimination method, the BMI (OR: 0.71; *p* = 0.007), ASA score (OR: 120; *p* = 0.040), CRP (OR: 1.05; *p* = 0.014), and presence of appendicoliths (OR: 318; *p* = 0.003) were predictive for advanced stages of AA. The overall model evaluation showed a strong predictive power (Pseudo R^2^ = 0.697; LH-ratio: *p* < 0.001) and an AUC of 0.955 on the ROC curve (Figure 5).

## 4. Discussion

The prevalence of appendicoliths in acute appendicitis receiving surgery was underestimated. In this study, appendicoliths have been shown to be more prevalent in AA than estimated in previous studies in which patients received surgery (71.4% vs. approximately 18–52%) [9,21]. Through intraoperative extracorporeal incision, we could increase the detection rate of appendicoliths with a sensitivity up to 90%. In the comparative analysis of UAA versus CAA, the diameter and the volume of appendicoliths were larger in the CAA group. Also, the diameter of the largest appendicoliths within a specimen was a predictive factor for CAA at a cutoff of approximately 2.5 mm. This value seems to be plausible for the development of critical obstruction considering an average luminal diameter of healthy vermiform appendix ranging from 1.32 to 2.32 mm at the appendicular base [22]. Similarly to previous studies, our data show that the combination of appendicoliths, CRP levels, ASA score, and BMI is highly predictive for advanced stages of disease such as gangrenous or perforated appendicitis with appendicoliths being the strongest risk factor. This may also be reflected by increased hospitalization in patients with appendicoliths.

Previous studies combined imaging such as ultrasound and CT scans, intraoperative palpation, and histopathological evaluation of the specimen to assess the prevalence of appendicoliths [9,20,21]. However, those studies did not cut directly into the resected vermiform appendix, thereby potentially missing appendicoliths. CT scans are limited with varying detection rates of 38.7% due to the detection of only radiopaque and bigger appendicoliths [17]. In fact, appendicoliths collected intraoperatively in our study varied in size and degree of calcification and were partially neither detected by CT scan nor ultrasound nor both. Furthermore, previous studies were performed retrospectively and relied on intraoperative palpation and documentation to assess the presence of appendicoliths, which seems to miss certain appendicoliths [9,20,21]. Edema of the appendicular wall in the state of acute inflammation hampers tactile sensation and negatively influences detection rates. The histopathological detection of fixed samples may miss non-calcified and smaller appendicoliths due to the exposure of appendicoliths to formalin aldehyde before they are investigated by the pathologist. Altogether, this may lead to an underestimation of the prevalence of appendicoliths. Indeed, in those studies, only 34% of appendicoliths were detected due to preoperative CT scan, intraoperative palpation and histopathological examination combined [9].

In the CODA study in 2021, with 776 of 1552 patients allocated to the conservative treatment arm, 25% of the patients experienced therapy failure without an appendicolith detected by imaging diagnostics [4]. Our data suggest that the rate of actual appendicoliths is higher than previously expected, particularly when compared to preoperative imaging. This masked rate of appendicoliths could explain some complicated courses of initially diagnosed uncomplicated appendicitis with therapy failure of non-surgical treatment of AA. However, being both an independent prognostic factor for the failure of non-operative treatments (NOT) of UAA and a risk factor for perforation, appendicoliths are considered criteria for performing an appendectomy [11]. Consequently, the safety of pretherapeutic diagnosis and the NOT approach should be critically reviewed.

Although our data provide new information for the prevalence of appendicoliths and their correlation with the severity of the disease, they underlie limitations. Our data give information about the presence of appendicoliths in adults only after the surgical procedure. As an important subgroup, patients not undergoing surgery could not be assessed. Also, children and adolescents were not included in the study, which is the reason for the limited sample size in a short period of time. Additionally, this study was performed monocentrically at one tertiary health care center in Germany and might be biased due to local factors.

## 5. Conclusions

To our knowledge, this was the first prospective study using the intraoperative incision of vermiform appendix. In this study, we were able to unmask the higher rate of appendicoliths and show its associations with severe stages of the disease and clinical course. The higher prevalence of appendicoliths may explain therapy failures of NOT in patients where appendicoliths were not found by routine imaging. Therefore, for better management of AA, more research is needed on methods that help to detect hidden appendicoliths.

## Figures and Tables

**Table 1:** Demographics and clinical characteristics of the patients with acute appendicitis with and without appendicoliths.

	Appendicolith Negative	Appendicolith Positive	p-Value (α = 0.05)
*n* (%) or mean (SD) or median (IQR)	16 (28.6)	40 (71.4)	
Demographics and Patient characteristics			
Gender			0.541
Female	7 (43.7)	14 (35.0)	
Male	9 (56.3)	26 (65.0)	
Age [14]	41.1 (±17.7)	48.4 (±18.6)	0.183
BMI [33]	27.4 (25.0; 31.7)	27.15 (25.0; 30.65)	0.717
ASA score	1.9 (±0.4)	2.1 (±0.7)	0.543
Diagnostics			
Markers of inflammation			0.435
Leucocyte count [10^9^/l]	13.4 (11.4; 18.0)	14.5 (12.7; 17.65)	0.416
CRP [mg/L]	24.9 (11.0; 58.2)	43.6 (11.4; 108.45)	0.320
Stages of disease according to EAES ^§^ classification			
Uncomplicated (Catarrhalic) Appendicitis	3 (18.75)	3 (7.5)	0.218
Complicated Appendicitis	13 (81.2)	37 (92.5)	
Phlegmonous	12 (75.0)	9 (22.5)	
Gangrenous	1 (6.2)	7 (17.5)	
Perforation ± abscess	0 (0.0)	21 (52.5)	

^§^ European Association of Endoscopic Surgery. ASA score: American Society of Anesthesiologists score; BMI: body mass index; CRP: C-reactive protein.

**Table 2:** Detection rates for appendicoliths by different diagnostic modalities.

	Appendicoliths Detected *n* (%)		
Modality	Yes	No	*p*-Value (α = 0.05)
Total number of patients	40 (71.4)	16 (28.6)	
Combined imaging	7 (12.5)	3 (5.3)	0.056
Ultrasound CT only	17 (30.3)	29 (51.8)	0.056
Ultrasound	5 (8.9)	23 (41.1)	0.239
Computer tomography (1 mm)	12 (21.4)	4 (7.1)	0.420
Preoperative imaging (CT and/or US)	24 (42.9)	32 (57.1)	0.001
Intraoperative examination	36 (64.3)	20 (35.7)	0.001
Histopathological examination	2 (3.6)	54 (96.4)	0.362

US: ultrasound; CT: computed tomography.

**Table 3:** The severity of acute appendicitis depends on the presence of appendicoliths.

Severity	Appendicolith Negative *n* = 16	Appendicolith Positive *n* = 40	*p*-Value (α = 0.05)
Simple AA (*n* = 27)—(%)	15 (26.8)	12 (21.4)	< 0.001
Catarrhalic	3 (5.3)	3 (5.3)	
Phlegmonous	12 (21.4)	9 (16.1)	
Advanced AA (*n* = 29)—(%)	1 (1.8)	28 (50.0)	<0.001
Gangrenous	1 (1.8)	7 (12.5)	
Perforation ± abscess	0 (0.0)	21 (37.5)	
Mean LOS (days)	5.19 (±3.7)	8.14 (± 5.4)	0.003
Complications (CD classification) ^§^			
Major compl. (CD ≥ 3) no.—(%)	0 (0.0)	6 (10.7)	0.124

^§^ CD: Clavien–Dindo classification of surgical complications, 2004 (1992); AA: acute appendicitis; LOS: length of stay.

**Table 4:** Logistic regression model including parameters to predict advanced stages of AA.

Parameter	OR	SE	Wald’s X^2^	*p*-Value (α = 0.05)	95% Conf. Interval	
Constant	0.09	0.24	6.88	0.380	0.000	20.21
Appendicoliths	318	614	47.8	0.003	7.258	13966
BMI	0.71	0.09	8.66	0.007	0.550	0.911
ASA score	119	28.2	7.19	0.040	1.144	332.2
CRP	1.05	0.02	6.74	0.014	1.010	1.097

## Data Availability

All data generated or analyzed during this study are included in this published article (and its Supplementary Materials File).

## Supplementary Materials

The following supporting information can be downloaded at: https://www.mdpi.com/article/10.3390/jcm13144166/s1, Figure S1. Flow chart of patients with suspected or diagnosed acute appendicitis (AA) with intraoperatively confirmed acute appendicitis with intraoperative incision. Twelve patients were excluded because of conservative treatment (*n* = 10) and false positive diagnosis of appendicitis (*n* = 2). Figure S2. The sample size calculation was based on the highest documented prevalence of appendicoliths from the previous literature (52%). In our study, we identified appendicoliths in 71.4% of patients with suspected or diagnosed appendicitis who underwent surgery (*n* = 40). The observed effect size was 19.4. Using an alpha level of 0.05, two-sided testing, and a statistical power of 0.8, we estimated that a sample size of 50 patients was necessary. Consequently, after analyzing preliminary data, we decided to conclude the study after including 56 patients. Table S1. Characteristics of appendicoliths differ between uncomplicated and complicated acute appendicitis (UAA and CAA). Table S2. A logistic regression model revealed the diameter of largest appendicoliths within one specimen to be predictive for complicated acute appendicitis (CAA).
