# Supplementary material for: Hidden Appendicoliths and Their Impact on the Severity and Treatment of Acute Appendicitis
Source: J Clin Med. 2024 Jul 16;13(14):4166. doi: 10.3390/jcm13144166 (PMC11278186; doi:10.3390/jcm13144166)
Supplement: Supplementary file 1 [file jcm-13-04166-s001.zip › jcm-3017440-supplementary.pdf]

Supplementary material

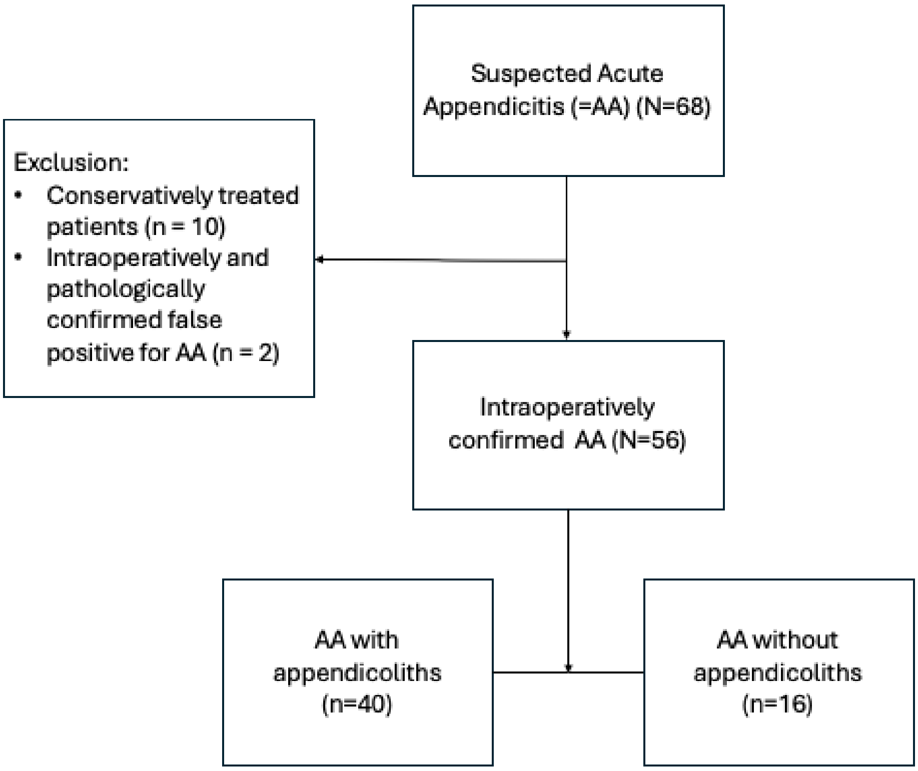

Supplemenatary Figure S1. Flow chart of patients with suspected or diagnosed acute appendicitis (AA) with intraoperatively confirmed acute appendicitis with intraoperative incision. Twelve patients were excluded because of conservative treatment (n = 10) and false positive diagnosis of appendicitis (n = 2).

Sample size calculation

```
. power oneproportion 0.52 0.714

Performing iteration ...

Estimated sample size for a one-sample proportion test
Score z test
H0: p = p0  versus  Ha: p != p0

Study parameters:

        alpha =    0.0500
        power =    0.8000
        delta =    0.1940
           p0 =    0.5200
           pa =    0.7140

Estimated sample size:

            N =        50
```

Supplementary Figure S2. The sample size calculation was based on the highest documented prevalence of appendicoliths from previous literature (52%). In our study, we identified appendicoliths in 71.4% of patients with suspected or diagnosed appendicitis who underwent surgery (n=40). The observed effect size was 19.4. Using an alpha level of 0.05, two-sided testing, and

a statistical power of 0.8, we estimated that a sample size of 50 patients was necessary. Consequently, after analyzing preliminary data, we decided to conclude the study after including 56 patients.

Supplementary Table S1. Characteristics of appendicoliths differ between uncomplicated and complicated acute appendicitis (UAA and CAA).

Characteristics of appendicoliths	UAA (SD) Mean (SD), median (IQR) or n (%)	CAA (SD) Mean (SD), median (IQR) or n (%)	p-value ($\alpha = 0.05$)
Number of appendicoliths	1 (1; 2)	2 (1; 3)	0.382
Diameter of largest apendicolith [mm]	0.62 (0.61; 2.45)	3.70 (2.50; 6.0)	0.016
Volume max. [mm^3]	1.0 (1.0; 62)	216.5 (72; 1288)	0.008
Volume cum. [mm^3].	52.5 (1.0; 104)	304.0 (111; 1664)	0.060

Supplementary Table S2. A logistic regression model revealed the diameter of largest appendicoliths within one specimen to be predictive for complicated acute appendicitis (CAA).

Parameter	OR	SE	Wald's X^2	p-value ($\alpha = 0.05$)	95% conf. interval	
Constant	0.10	0.183	1.64	0.200	0.003	3.313
Diameter of appendicoliths	7.87	7.27	4.99	0.025	1.288	48.14
